# Using ^13^C isotopes to explore denitrification-dependent anaerobic methane oxidation in a paddy-peatland

**DOI:** 10.1038/srep40848

**Published:** 2017-01-18

**Authors:** Yao Shi, Zhongqiang Wang, Chunguang He, Xinyu Zhang, Lianxi Sheng, Xiaodong Ren

**Affiliations:** 1 State Environment Protection Key Laboratory of Wetland Ecology and Vegetation Restoration, Northeast Normal University, Changchun 130024, China; 2 School of Environment, Northeast Normal University, Changchun 130024, China; 3 School of Geographical Sciences, Northeast Normal University, Changchun 130024, China; 4 Key Laboratory of Ecosystem Network Observation and Modeling, Institute of Geographic Sciences and Natural Resources Research, Chinese Academy of Sciences, Beijing 100101, China; 5 Key Laboratory for Molecular Enzymology and Engineering of Ministry of Education, Jilin University, Changchun 130023, PR China

## Abstract

Peatlands are organic-matter-rich but nitrogen-limited natural systems, the carbon/nitrogen (C/N) status of which are subject to increasing exposure from long-term nitrate (NO_3_^−^) fertilizer inputs and atmospheric nitrogen (N) deposits. To manage and protect these unique environments, an improved understanding of denitrification-dependent anaerobic oxidation of methane (DAMO) in peatlands is needed. In this study, we used stable isotope measurements and incubation with NO_3_^−^ additions to facilitate an investigation and comparison of the potential DAMO rates in a paddy-peatland that has been influenced by N fertilizer over 40 years and an undisturbed peatland in northeast China. Monitoring of ^13^CO_2_ production confimed DAMO did occur in both the paddy-peatland and the undisturbed peatland, the rates of which increased with NO_3_^−^ additions, but decreased logarithmically with time. When NO_3_^−^ was added, there were no significant differences between the CH_4_ oxidation in the paddy-peatland and peatland samples after 36 hours of incubation (97.08 *vs*. 143.69 nmol g^−1^ dry peat) and the potential DAMO rate after incubation for 1 hour (92.53 *vs*. 69.99 nmol g^−1 ^h^−1^). These results indicate that the occurrence of DAMO in peatlands might be controlled by the amount of NO_3_^−^ applied and the depth to which it penetrates into the anoxic layer.

Anaerobic oxidation of methane (AOM) is an important process that controls methane in marine and freshwater ecosystems, which may couple with sulfate (SO_4_^2−^)^1^, ferric iron (Fe^3+^)^2^ and nitrite (NO_2_^−^)/nitrate (NO_3_^−^) reduction^3^. Of these pathways, denitrification-dependent anaerobic oxidation of methane (DAMO), also known as NO_3_^−^/NO_2_^−^ dependent anaerobic methane oxidation, can mitigate CH_4_ and N_2_O emissions simultaneously (Equation (1)). Set in the context of increasing atmospheric N deposition and applications of N fertilizers to natural ecosystems (Fig. 1), this process is rapidly gaining interest as a key issue in global C and N cycles.


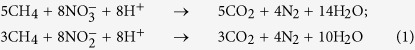


Researchers have observed DAMO in NO_3_^−^-rich sediment from a freshwater pond^4^, Lake Constance^5^, and wetland sediment^6^. Norði *et al*.^4^ demonstrated that DAMO was considerably enhanced in a freshwater sediment enriched by nitrate (1–2 mmol L^−1^). Deutzmann *et al*.^5^ concluded that, if NO_3_^−^ was available in anoxic zones, the DAMO process could completely eliminate methane emissions from sediments in freshwater environments. The different effects of NO_3_^−^ additions on the potential DAMO rates are probably related to the soil properties and the amount of NO_3_^−^ added. While freshwater systems are C-limited, northern peatlands have accumulated about one third of global soil C as partially decomposed organic matter^7^ and emit about 30 Tg CH_4_ annually^8^. Although some attention has been directed to the mechanisms that control CH_4_ transformations in peatlands, our understanding of this process is incomplete. Freshwater and marine systems, with low-organic matter contents, contrast starkly with peatlands, which are CH_4_-rich ecosystems with organic matter throughout their entire profile. However, DAMO in the anoxic layer of peatlands has long been overlooked and seems unlikely to happen, as NO_3_^−^ availability is limited because of inadequate N inputs, low nitrification^9^, or rapid denitrification^10^. Various researchers have added ^13^C-CH_4_ under anaerobic conditions and have measured ^13^CO_2_ after incubation to confirm the occurrence of DAMO and to quantitatively estimate the amount of oxidized CH_4_^5^,^6^,^11^,^12^,^13^. Smemo and Yavitt^13^, who used stable isotopes (^13^C-CH_4_) in electron acceptor experiments, confirmed the occurrence of DAMO and suggested that further research should focus on DAMO in peatlands. Zhu *et al*.^11^ reported that there was microbial evidence for the DAMO process in the anoxic layer of a minerotrophic peatland in the Netherlands that was subject to infiltration of NO_3_^−^-rich groundwater. They suggested that DAMO may have been an important mechanism for CH_4_ consumption under increased NO_3_^−^ levels in the anoxic layer of peatlands. However, Gupta *et al*.^14^ reported that, based on their study in Michigan Hollow (a minerotrophic fen in the US), NO_3_^−^ additions did not cause induced DAMO. These contrasting results suggest that the effects of NO_3_^−^ additions on the DAMO process in peatlands are not well-defined and deserve further investigation. Therefore, to gain an improved understanding of the mechanisms that control the occurrence of DAMO, the influence of long-term N inputs on the DAMO process needs to be studied.

In northeast China, many peatlands have been converted to paddy fields, and rice is now cultivated in the topsoil. The topsoil is underlain by a layer of peat, thereby forming a unique profile known as “a paddy-peatland” field. In the paddy-peatland system, increased amounts of NO_3_^−^ penetrate the anoxic layer because of N fertilization year after year. This environment therefore provides the opportunity for methane and NO_3_^−^ to meet and interact at the anoxic layer, thereby forming favorable conditions for the DAMO process. Taking advantage of this environment, the aim of the present study was to investigate the occurrence of DAMO in paddy-peatland soils under long-term NO_3_^−^ fertilization. We selected two sampling sites, one in a fertilized paddy-peatland and the other in an undisturbed peatland, in the Jinchuan Peatland in northeast China. We also conducted an NO_3_^−^ addition incubation experiment using ^13^CH_4_ isotope-labeling measurements to determine whether or not DAMO occurred and to ascertain if NO_3_^−^ additions stimulated anaerobic CH_4_ oxidation and DAMO rates in the paddy-peatland soils.

## Results

### Physical and chemical parameters

The chemical properties of peat and porewater samples collected from the paddy-peatland and peatland at the beginning of the trials are summarized in Table 1. The NO_3_^−^ and organic carbon concentrations in the peat samples from the paddy-peatland and peatland were similar, but there were significant differences between the porewater pH, NO_3_^−^, and DOC concentrations of the paddy-peatland and peatland soils (*P* < 0.05). The pH ranged from 5.27 to 5.51 in the paddy-peatland and was significantly lower than the range (from 5.88 to 6.07) in the peatland. The porewater NO_3_^−^ concentrations were significantly higher in the paddy-peatland (from 0.047 to 0.081 mg L^−1^) than in the peatland (from 0.003 to 0.027 mg L^−1^). Also, the DOC concentrations in the paddy-peatland were markedly higher than in the peatland, and varied from 35.10 to 36.06 mg L^−1^ in the paddy-peatland and from 25.83 to 31.87 mg L^−1^ in the peatland (Table 1).

### Evidence for DAMO occurrence

**Figure 2:**
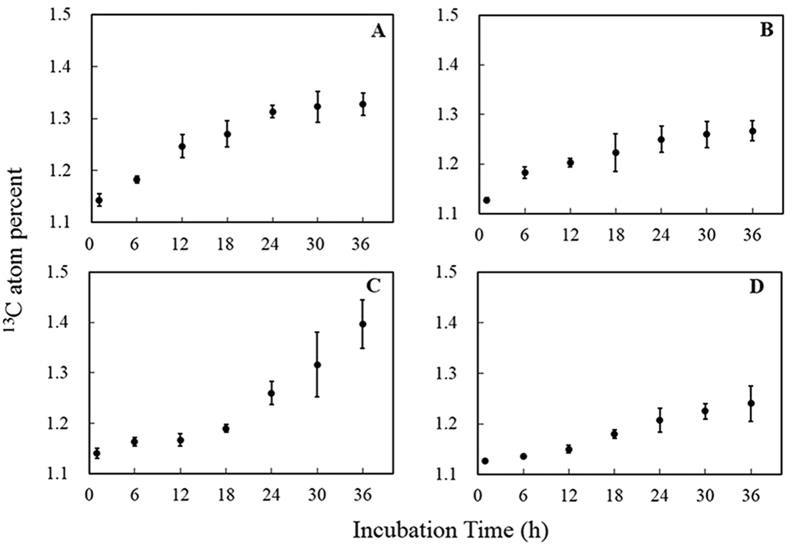
Variations in the ^13^CO_2_ atom percentage over the incubation time (h). The ^13^CO_2_ was produced by peat samples from the peatland and paddy-peatland incubated with four treatments: (**A**) ^13^CH_4_ (PC), (**B**) ^13^CH_4_ + NO_3_^−^ (PN), (**C**) ^13^CH_4_ (PPC), and (**D**) ^13^CH_4_ + NO_3_^−^ (PPN). PC refers to peatland samples with ^13^CH_4_ additions; PN refers to peatland samples with ^13^CH_4_ + NO_3_^−^ additions; PPC refers to paddy-peatland samples with ^13^CH_4_ additions, and PPN refers to paddy-peatland samples with ^13^CH_4_ + NO_3_^−^ additions.

Peat samples taken from the paddy-peatland and peatland were able to oxidize CH_4_ anaerobically. There were similar trends in the percentages of the headspace ^13^CO_2_ atoms percent (^13^CO_2_ AT%) in the paddy-peatland PPN and PPC treatments, and the peatland PC and PN treatments. The ^13^CO_2_ AT% increased significantly and positively during the incubation time in the PPN, PPC, PC, and PN treatments (*P* < 0.05; Fig. 2), which suggests that DAMO occurs in the peat samples from the paddy-peatland and the peatland.

**Figure 3:**
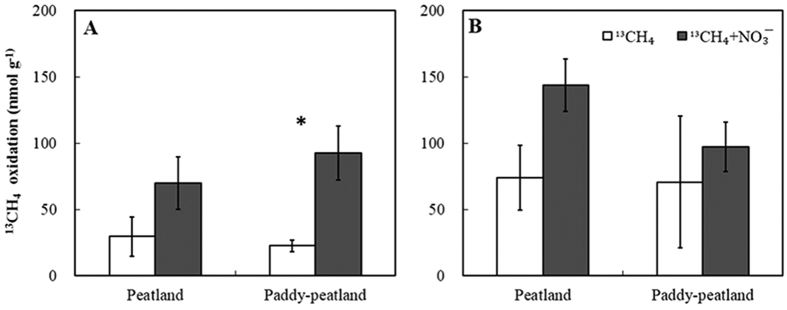
Average ^13^CH_4_ oxidation in the PPC, PPN, PC, and PN treatments after incubating for 1 hour (**A**) and 36 hours (**B**). The vertical bars represent the standard error of the mean. Asterisks indicate the level of significance (*P < 0.05; *n* = 4). PPC refers to paddy-peatland samples with ^13^CH_4_ addition, PPN refers to paddy-peatland samples with ^13^CH_4_ + NO_3_^−^ additions, PC refers to peatland samples with ^13^CH_4_ additions, and PN refers to peatland samples with ^13^CH_4_ + NO_3_^−^ additions.

The average ^13^CH_4_ oxidation in the peat samples from the paddy-peatland and the peatland after 1 hour and 36 hours of incubation are presented in Fig. 3. The average ^13^CH_4_ oxidation was much higher in paddy peatland soils to which ^13^CH_4_ and NO_3_^−^ had been added at *t* = 1 h than in the samples to which only ^13^CH_4_ was added. After incubating for 36 hours (*t* = 36 h), there was no significant difference between the average ^13^CH_4_ oxidation in the paddy-peatland samples to which ^13^CH_4_ and NO_3_^−^ was added (Fig. 3B) and the paddy-peatland samples to which only ^13^CH_4_ was added. The effects of ^13^CH_4_ and NO_3_^−^ additions were more pronounced at *t* = 1 h (Fig. 3A). The average ^13^CH_4_ oxidation was 3.1 and 0.38 times greater in the paddy-peatland samples than in the dry peat at *t* = 1 h (92.53 *vs*. 22.38 nmol g^−1^ dry peat) and *t* = 36 h (97.08 *vs*. 70.50 nmol g^−1^ dry peat), respectively.

### The potential DAMO rates

**Figure 4:**
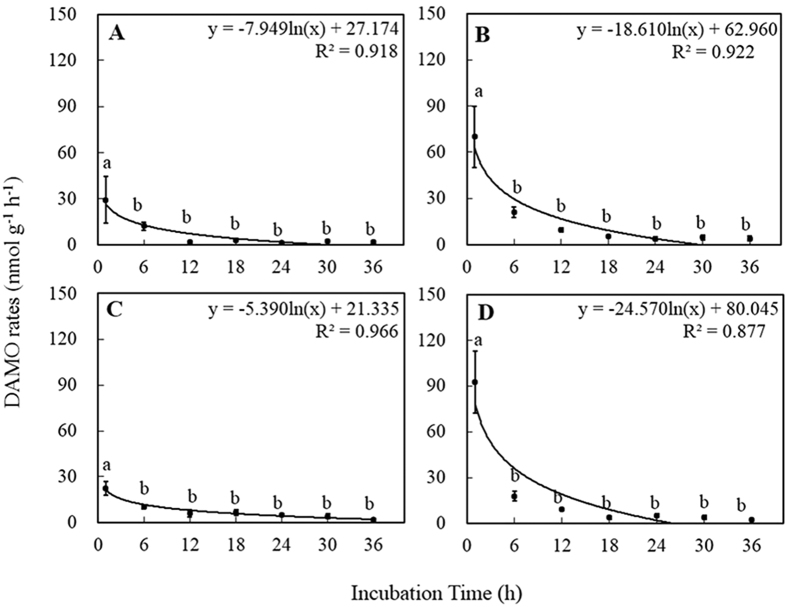
Scatter plots of incubation and DAMO rates over 36 h in peat samples collected from the two sampling sites incubated with four treatments: (**A**) ^13^CH_4_ (PC), (**B**) ^13^CH_4_ + NO_3_^−^ (PN), (**C**) ^13^CH_4_ (PPC), and (**D**) ^13^CH_4_ + NO_3_^−^ (PPN). Different lower case letters indicate significant differences within a given time frame. The vertical bars represent the standard error of the mean (*n* = 4). The potential DAMO rates were calculated from nonlinear regression of the concentrations of ^13^CO_2_ produced in the headspace of the vial over time. PC refers to peatland samples with ^13^CH_4_ additions; PN refers to peatland samples with ^13^CH_4_ + NO_3_^−^ additions; PPC refers to paddy-peatland samples with ^13^CH_4_ additions; PPN refers to paddy-peatland samples with ^13^CH_4_ + NO_3_^−^ additions.

In this study, the potential DAMO rates in the paddy-peatland and peatland decreased logarithmically with incubation time (Fig. 4). The potential DAMO rates were significantly greater at *t* = 1 h than at *t* = 6, 12, 18, 24, 30, and 36 h in both the paddy-peatland and peatland soils (*P* < 0.05). The potential DAMO rates of the paddy-peatland and peatland samples were 4.1 (92.53 *vs*. 22.38 nmol g^−1 ^h^−1^) and 2.4 times (69.99 *vs*. 29.35 nmol g^−1 ^h^−1^) greater in the samples to which NO_3_^−^ was added at *t* = 1 h than in the peat samples to which only ^13^CH_4_ was added. After 36 hours’ incubation, the potential DAMO rate was higher for PPN than for PPC. However, there was no significant difference between the potential DAMO rates in the PPN and PPC treatments (2.29 nmol g^−1 ^h^−1^ *vs*. 1.96 nmol g^−1 ^h^−1^) after incubation for 36 hours. As for the paddy-peatland, the potential DAMO rates of PN were higher than those of PC. The potential DAMO rates for PN and PC (3.99 nmol g^−1 ^h^−1^ *vs*. 12.05 nmol g^−1 ^h^−1^) were not significantly different at the end of the incubation experiment (*t* = 36 h).

The ^13^CH_4_ oxidation and potential DAMO rates increased significantly after additions of NO_3_^−^ to both the paddy-peatland and peatland soils at *t* = 1 h (*P* < 0.01; Table 2). However, the ^13^CH_4_ oxidation and potential DAMO rates were not significantly different from those of the soil without NO_3_^−^ additions after incubation for 36 hours (*P* < 0.05). The ^13^CH_4_ oxidation and potential DAMO rates at both *t* = 1 h and *t* = 36 h were not significantly different between the paddy-peatland and peatland. There was no significant difference in the interaction effects between sampling sites and treatments.

## Discussion

### Occurrence of DAMO

The results from our study showed that DAMO occurred in both the paddy-peatland and peatland samples over the entire incubation time (under a temperature of 18 °C). Under anaerobic incubation conditions, ^13^CO_2_ was produced by the paddy-peatland and peatland samples, which verified that the headspace ^13^CO_2_ came from ^13^CH_4_ oxidation^12^,^14^. Our results confirm that DAMO occurred in the paddy-peatland samples, and that it commenced immediately after NO_3_^−^ was added in the first hour. Potential DAMO rates declined significantly with time, which showed that the C and N availablility were key controls on the occurrence of DAMO in the anoxic layer. Peatland soils are high in organic matter; therefore the N concentration determines the occurrence of DAMO. Given that the NO_3_^−^ turnover via denitrification in peat soils is rapid^15^, and that the growth of DAMO bacteria should be taken into consideration when estimating the potential DAMO rate in long-term incubations^6^, we carried out an incubation experiment over a period of 36 hours to examine the effect of NO_3_^−^ additions on DAMO rates in peat samples. Very few studies have previously reported the potential DAMO rates^12^,^13^,^14^, and most of these existing studies have reported potential DAMO rates over an incubation time of weeks or months, which is much longer than the incubation time in our study (36 hours). For example Zhu *et. al.*^11^ determined the NO_2_^−^-dependent anaerobic methane oxidation activity in a minerotrophic peatland with additions of ^13^CH_4_ and NO_2_^−^ over an incubation period of 3 months. They did not detect any NO_2_^−^-dependent anaerobic methane oxidation in the first two weeks of the incubation experiment, which suggests that the NO_2_^−^-dependent anaerobic methane oxidation activity was the result of DAMO bacteria enrichment. However, Hu *et al*.^6^, in their recent short-term incubation experiment on NO_2_^−^-dependent anaerobic methane oxidation activity, reported that NO_2_^−^-dependent anaerobic methane oxidation occurred in wetland soils after incubations of 20 hours when ^13^CH_4_ and NO_3_^−^ were added.

### Differences in DAMO rates

In our experiment, the potential DAMO rates at *t* = 1 h were significantly higher than those at *t* = 6, 12, 18, 24, 30, and 36 h for both the paddy-peatland and peatland samples. The enhanced DAMO rates when the NO_3_^−^ additions started may reflect a priming effect. Mineralization of soil organic matter may be accelerated or retarded when organic substrates are added^16^. We also observed a significant increase in the potential DAMO rates when NO_3_^−^ was added to the paddy-peatland and peatland samples at *t* = 1 h (*P* < 0.05), which suggests that the DAMO process was limited by the amount of available NO_3_^−^ or NO_2_^−^ in the two sampling sites. In the present study, the NO_3_^−^ concentrations in peat samples taken from the peatland and paddy-peatland were 0.24 and 1.63 mg kg^−1^, respectively (Table 1), which are both much lower than those reported by Gauthier *et al*.^17^ (12–17 mg kg^−1^). It has been reported that peatlands are often limited by available N because of the fast N turnover rates^15^. Therefore, NO_3_^−^ additions increased the amount of available NO_3_^−^ and resulted in enhanced DAMO rates at the beginning of the incubation experiment (*t* = 1 h). DAMO rates decreased as the available NO_3_^−^ was gradually depleted during the incubation. The potential DAMO rates after NO_3_^−^ additions after 36 hours incubation at 18 °C (*t* = 36 h) in our study were lower than those reported by Gupta *et al*.^14^, who reported a rate of 5.94 nmol g^−1 ^h^−1^ (at 19 °C) when *t* = 21 days, but were higher than those reported by Zhu *et al*.^11^ (0.38 nmol g^−1 ^h^−1^ at 25 °C when *t* = 3 months). Without NO_3_^−^ additions, the potential DAMO rates at *t* = 1 h were higher than those reported by Smemo and Yavitt^13^ (61.2 nmol g^−1 ^h^−1^) after a 4-year incubation and by Blazewicz *et al*.^12^ (0.88 nmol g^−1 ^h^−1^) after a 71-day incubation (both at 25 °C). They found that NO_3_^−^ had no effect on DAMO in peat samples. These different results probably result from the occurrence of multiple concurrent AOM pathways at a given time in peatlands^12^,^14^,^17^.

Previous research has shown that DAMO rates are significantly enhanced by NO_3_^−^, and that DAMO can consume large quantities of CH_4_ from NO_3_^−^-rich environments, with previous studies reporting consumption rates of between 31 to 437 μmol CH_4 _m^−2 ^d^−1^ 4,5. However, the peatlands contain much more organic carbon and have a much higher C/N than freshwater sediments. Thus, if NO_3_^−^ and CH_4_ interact in anoxic conditions in peatlands, the DAMO process will likely be induced. In addition, the effect of NO_3_^−^ on DAMO rates is probably related to the peat/sediment properties and the amount of NO_3_^−^ added.

In this study, ^13^CH_4_ oxidation and the potential DAMO rates at *t* = 1 h and *t* = 36 h in the paddy-peatland and peatland samples were not significantly different, while NO_3_^−^ additions resulted in enhanced ^13^CH_4_ oxidation and potential DAMO rates at *t* = 1 h (*P* < 0.05; Table 2). In general, NO_3_^−^ concentrations should be high in the paddy-peatland samples because of N fertilizer applications. Zhang *et al*.^18^ previously reported high NO_3_^−^ concentrations in a paddy field, with much higher concentrations close to the surface than deeper in the peat profile^19^,^20^. However, NO_3_^−^ concentrations were not elevated in our samples, which may reflect the depth from which we collected the samples (50–60 cm below the soil surface). Nitrification generally occurs under oxic conditions, which most likely occurred in the upper layer of the paddy-peatland. As NO_3_^−^ moves into the deeper layers by leaching, it may be transformed or consumed by plants and microbes^21^. The depth at which DAMO occurs needs to be examined further, as it may be possible to detect significant differences in the ^13^CH_4_ oxidation and DAMO rates between the paddy-peatland and peatland at less than 50 cm deep in the anoxic layer. Further, ^13^CH_4_ oxidation rates were similar in the paddy-peatland and peatland similar, and may reflect the fact that we collected the peat samples at the end of the growing season, at which time the available N in the paddy-peatland soils may have been either used for rice growth or mineralized by microbes.

## Conclusion

This study provides direct evidence of DAMO in paddy-peatland samples that had been in receipt of NO_3_^−^ fertilizer inputs for over 40 years. The results of our incubation experiments showed that the potential DAMO rates decreased logarithmically as the incubation time increased. There was no significant difference between the anaerobic CH_4_ oxidation and the potential DAMO rates in the paddy-peatland and peatland samples. But CH_4_ oxidation and the potential DAMO rates increased as NO_3_^−^ was added. This indicated that in peatlands, the occurrence of DAMO may depend on the amount of NO_3_^−^ and the depth to which it penetrates into the anoxic layer, the area where increased downward fluxes of NO_3_^−^ and upward fluxes of methane meet. However, DAMO is a relatively complex process, and further investigations, including consideration of the vertical distribution of DAMO-associated microorganism species in peatlands, and response to environmental factors, such as temperature, pH and redox potential are needed to obtain a better understanding in peatlands.

## Materials and Methods

### Study area

**Figure 5:**
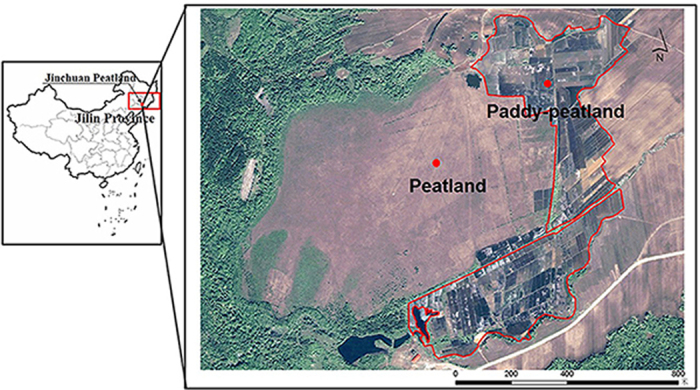
The two sampling sites in the Jinchuan Peatland. (The Jinchuan peatland information was generated from the remote sensing images, and it was edited with ArcGIS 10.2 software, http://www.esrichina.com.cn/softwareproduct/ArcGIS/).

We chose a study area in a peatland at Jinchuan, Jilin Province, Northeast China (42°20′56″N, 126°22′51″E) (Fig. 5). The peatland is classified as a minerotrophic fen. It extends over a total area of 0.986 km^2^ and its elevation ranges from 613 to 616 m. The peat formed about 7000 years ago, during the early Holocene Epoch, and is between 4 and 6 m deep. The area is dominated by a temperate continental monsoon climate. The mean annual precipitation is about 1054 mm. The water on the surface of the peatland is normally about 10 cm deep in July and August. The vegetation is dominated by *Carex schmidtii, C. tenuiflora, Phragmites australis–Carex spp*., *C. schmidtii–Sphagnum spp*., *Carex spp*.*–Deyeuxia angutifolia*, and *Betula ovalifolia–Sphagnum spp*.

An area of about 0.37 km^2^ in the eastern and southern parts of the Jinchuan peatland has been reclaimed and has been used for agriculture (mainly rice cultivation) since the 1970 s (Fig. 5). The cultivated topsoil is underlain by a peat layer (60–80 cm).

### Field sampling

To examine the effects of N inputs on the DAMO process, peat samples (50–60 cm below the peat surface) were collected from two sampling sites in September 2015, namely a long-term fertilized rice field (referred to as paddy-peatland) in the northeast of the Jinchuan peatland, and an undisturbed peat field (referred to as peatland) as a control (Fig. 5). On collection, peat samples were immediately put into plastic vacuum self-sealed bags and the excess air was removed by suction to minimize exposure to O_2_. All samples were transported to the laboratory within 24 h of collection in a cool box with ice and stored at 4 °C until analysis.

### Isotope Tracer Experiments

1. **Pre-incubation:** The peat samples were pre-incubated at room temperature (18 °C) in the dark for 12 days to remove any residual NO_x_^−^ and O_2_ and to ensure that the samples were completely anoxic;
2. **Sample division:** Wet samples of peat (50 g) and 50 ml of sterile deionized H_2_O were transferred into 500 ml culture vials and sealed with screw-cap lids in an anoxic glove box;
3. **Treatments:** The peat samples were subsequently divided into four treatment groups: (1) paddy-peatland soils with additions of ^13^CH_4_ (^13^C at 99.9%), referred to as PPC; (2) paddy-peatland soils with additions of ^13^CH_4_ + NO_3_^−^, referred to as PPN; (3) peatland soils with ^13^CH_4_ added (^13^C at 99.9%), referred to as PC; (4) peatland soils with ^13^CH_4_ + NO_3_^−^ added, referred to as PN.
4. **Addition of NO**_**3**_^−^ **and** ^**13**^**CH**_**4**_: The headspace gas in vials was checked for residual methane and carbon dioxide. When undetectable, KNO_3_ solution (0.1 mM, 50 mL) and ^13^CH_4_ (10 mL) were injected into vials through the silicone septa in anoxic glove box^14^, resulting in a final methane concentration of 2.5 × 10^4^ mg m^−3^ in the headspace of each vial;
5. **Gas sampling:** The headspace atmosphere (200 mL) in each vials were sampled after 1, 6, 12, 18, 24, 30, and 36 hours of incubation, with a needle and syringe and injected into a pre-evacuated polyethylene-coated aluminum bag. Four replicates of each treatment were set up at different sampling times, and there were a total of 112 samples.

### Analytical Methods

The headspace gas of each sampling vial was analyzed for *δ*^13^C-CO_2_ PDB (Pee Dee Belemite) using an isotope mass spectrometer (MAT253GC-IRMS, Thermo Finnigan, USA).

The peat water content was determined as the weight loss from wet peat dried at 105 °C for 24 h. The peat organic carbon concentration was determined using the K_2_Cr_2_O_7_ oxidation method. Ammonium and NO_3_^−^ were extracted from the peat soil using 2 M KCl. The concentrations of total nitrogen (TN), ammonium (NH_4_^+^), and NO_3_^−^ in the peat were determined using a continuous flow analyzer (San^++^, Skalar, Breda, Netherlands). Wet peat was centrifuged at 4000 g for 10 minutes to extract the porewater. The supernatant was filtered through 0.45 μm cellulose acetate filters. The pH in porewater was determined using a pH meter. Porewater NO_3_^−^, NO_2_^−^, and NH_4_ concentrations were determined by a continuous flow analyzer (San^++^, Skalar, Breda, The Netherlands). Dissolved total carbon (DTC) and dissolved inorganic carbon (DIC) were determined with a C/N analyzer (Multi C/N 3000 Analyzer, Analytik Jena AG, Thuringia, Germany). Dissolved organic carbon (DOC) was calculated by subtracting DIC from DTC.

### Calculations and Data Analyses

Headspace ^13^CO_2_ production rates were calculated from the GC measurements of headspace gas and were expressed per mass of dry peat, as outlined by Gupta *et al*.^13^. The average ^13^C atom percent (AT%) of the four replicates from each treatment and time interval were then corrected for CO_2_ in the dissolved phase using Henry’s Law to determine how much headspace CO_2_ was unaccounted for because of equilibration with porewater^11^.

Statistical analyses were conducted using SPSS version 22.0 software (SPSS Inc., Chicago, IL, USA). One-way ANOVA was used to determine significant differences (*P* < 0.05) between treatments at the respective sampling times. An independent-samples *t* test was used to characterize the differences between the two sampling points. The average ^13^CO_2_ AT% was calculated by linear regression of the concentration of ^13^CO_2_ produced in the headspace of the vial per gram of dry peat. For isotopic analysis, *δ*^13^C PDB values were converted to the percentage of ^13^C atom (^13^C AT %) using equation (2), and the DAMO rate and CH_4_ oxidation were calculated using equations (3) and (4):


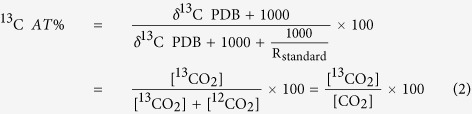



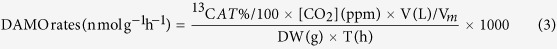



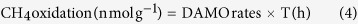


Where,

R_standard_ = 0.0112372^14^,

V = 0.2 L, V_m_ = 22.4 L/mol,

DW = Dry weight of peat samples (g),

T = Time of incubation (h).

## Additional Information

**How to cite this article**: Shi, Y. *et al*. Using ^13^C isotopes to explore denitrification-dependent anaerobic methane oxidation in a paddy-peatland. *Sci. Rep.* **7**, 40848; doi: 10.1038/srep40848 (2017).

**Publisher's note:** Springer Nature remains neutral with regard to jurisdictional claims in published maps and institutional affiliations.

## Figures and Tables

**Figure 1:**
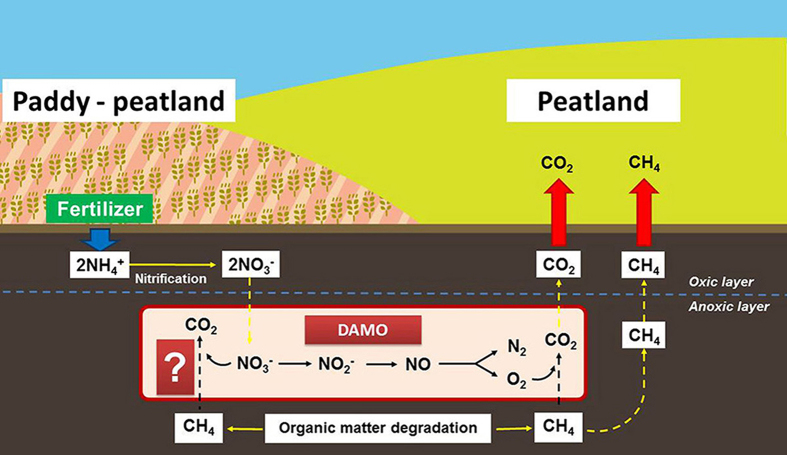
Conceptual diagram of greenhouse emissions in the paddy-peatland and peatland (Original picture drawn by Yao Shi, and the DAMO process pathway was referenced from Fig. 1(c) of Ettwig, *et al*.^22^).

**Table 1:** The chemical properties of peat samples and porewater (mg L^−1^) at 50–60 cm below the peat surface in the paddy-peatland and peatland.

Sampling point	Peat samples		Porewater samples		
	C-org %	NO_3_^−^ mg kg^−1^	pH	NO_3_^−^ mg L^−1^	DOC mg L^−1^
Peatland	40.42 (2.58)	0.24 (0.03)	**5**.**99** (**0**.**06**)**	**0**.**016** (**0**.**01**)*	**28**.**73** (**1**.**75**)*
Paddy-peatland	34.78 (1.37)	1.63 (0.37)	**5**.**40** (**0**.**07**)**	**0**.**064** (**0**.**01**)*	**35**.**58** (**0**.**28**)*

Significant differences between the paddy-peatland and peatland are indicated in bold; asterisks indicate the level of significance (*P < 0.05, **P < 0.01), *n* = 3. Mean values are shown with the standard error in brackets.

**Table 2:** The effects of sampling point, treatment, and point × treatment on DAMO rates and headspace ^13^CH_4_ oxidation.

	^13^CH_4_ oxidation (n mol g^−1^)		DAMO rates (n mol g^−1 ^h^−1^)	
	0–1 h	0–36 h	0–1 h	0–36 h
Sampling site	0.686	0.443	0.639	0.307
Treatment	**0**.012**	0.157	**0**.006**	0.202
Point × Treatment	0.898	0.503	0.380	0.358

Significant *p*-value of sampling point, treatment, and point × treatment are indicated in bold. Asterisks indicate the level of significance (**P < 0.01), *n* = 4.
